# Development of Activity and Participation Norms among General Adult Populations in Taiwan

**DOI:** 10.3390/ijerph14060603

**Published:** 2017-06-06

**Authors:** Chia-Feng Yen, Tzu-Ying Chiu, Tsan-Hon Liou, Wen-Chou Chi, Hua-Fang Liao, Chung-Chao Liang, Reuben Escorpizo

**Affiliations:** 1 Department of Public Health, Tzu Chi University, Hualien 97004, Taiwan; mapleyeng@gmail.com; 2 Institute of Health Policy and Management, National Taiwan University, Taipei 10055, Taiwan; yin827@gmail.com; 3 Department of Physical Medicine and Rehabilitation, Shuang Ho Hospital, Taipei Medical University, Taipei 23561, Taiwan; peter_liou@s.tmu.edu.tw; 4 Graduate Institute of Injury Prevention and Control, College of Public Health, Taipei Medical University, Taipei 11031, Taiwan; 5 Department of Physical Medicine and Rehabilitation, School of Medicine, College of Medicine, Taipei Medical University, Taipei 11031, Taiwan; 6 Department of Occupational Therapy, Chung Shan Medical University, Taichung 40201, Taiwan; 7 School and Graduate Institute of Physical Therapy, College of Medicine, National Taiwan University, Taipei 10051, Taiwan; hfliao@ntu.edu.tw; 8 Department of Physical Medicine and Rehabilitation, Buddhist Tzu Chi General Hospital, Hualien 97002, Taiwan; STONE@tzuchi.com.tw; 9 School of Medicine, Tzu Chi University, Hualian 97004, Taiwan; 10 Department of Rehabilitation and Movement Science, College of Nursing and Health Sciences, University of Vermont, Burlington, VT 05401, USA; escorpizo.reuben@gmail.com; 11 Swiss Paraplegic Research, Nottwil 6207, Switzerland

**Keywords:** activity and participation, functioning, disability, norms, WHODAS 2.0

## Abstract

Based on the International Classification of Functioning, Disability, and Health (ICF) and the World Health Organization Disability Assessment Schedule 2.0 (WHODAS 2.0), The Functioning Disability Evaluation Scale-Adult version (FUNDES-Adult) began development in 2011. The FUNDES-Adult was designed to assess the difficulty level of an individual’s activities and participation in daily life. There is a lack of research regarding the profile of activity and participation for the general adult population. The purposes of this study were to establish activity and participation norms for the general adult population in Taiwan and to describe, discuss, and compare the activity and participation profile with other population. Method: A population-based survey was administered in 2013 using a computer-assisted telephone interviewing system (CATI system). Using probability proportional to size (PPS) sampling and systematic sampling with random digit dialing (RDD), 1500 adults from Taiwan’s general population were selected to participate in the survey. The FUNDES-Adult with six domains and two dimensions (performance and capability) was used to obtain data on activities and participation levels. A higher domain score indicated higher participation restriction. Results: Approximately 50% of the respondents were male, and the average age of the respondents was 45.23 years. There were no significant differences in the demographic features between the sample and the population. Among the six domains, the self-care domain score was the lowest (least restriction) and the participation domain score was the highest (most restriction). Approximately 90% of the sample scored were less than 15, and only 0.1% scored more than 80. This is the first cross-national population-based survey to assess norms of activity and participation relevant to the general population of Taiwan. As such, the results of this survey can be used as a reference for comparing the activity and participation (AP) functioning of other countries and subgroups.

## 1. Introduction

In 2001, the International Classification of Functioning, Disability, and Health (ICF) was announced and released by the World Health Organization (WHO), which rapidly became a guiding model for disability research and a key tool for both a population-based and a clinical-based understanding of disability [1,2,3]. The ICF offers a biopsychosocial model that provides a conceptual framework linking body functions and structures with activities and participation [4]. In recent years, there has been an increase in interest regarding activity and participation outcomes as important objectives for healthy aging and for effective nonpharmacological intervention in the prevention and management of various diseases such as dementia and stroke as well as chronic conditions in children [5,6]. The assessment of activity and participation has not only been used for evaluations in clinical trials worldwide, it has also been incorporated into the current social security policy and special education act in the European Union and Portugal [7,8].

In 2007, Taiwan promulgated the People with Disabilities Rights Protection Act to enhance the social participation of people with disabilities. This act resulted in a regulation that requires the Disability Evaluation System to include an activities and participation component. Hence, the Taiwan ICF research team developed the Functioning Disability Evaluation Scale (FUNDES), which was modified from the 36-item version of the WHO Disability Assessment Schedule 2.0 (WHODAS 2.0) [9,10,11,12], thus providing a structured method for measuring the health and disabilities of adults (age ≥ 18 years) across cultures. The WHODAS 2.0 was translated into more than 30 languages between 2001 and 2010, and the traditional Chinese version of the WHODAS 2.0 was developed in 2011 [12,13]. Domains 1 through 6 of the FUNDES-Adult are as follows: cognition, mobility, self-care, getting along, life activities and participation. [12,13].

In Taiwan, the activity and participation levels for people with disabilities are assessed by the Disability Evaluation System. From this system we have created a decision support system to develop a disability determination model [14]. Such a disability determination model will facilitate the understanding of the association between impairment and the functioning levels with respect to activities and participation among those with disabilities. However, as there is a lack of research regarding the activity and participation profile for the general population in Taiwan, it is difficult to predict resource allocations to meet the needs of and provide the social support for people with disabilities. 

The purposes of this study were to establish the general adult population's activity and participation norms in Taiwan and to describe, discuss, and compare the activity and participation profile with other populations, which can be used as a reference to analyze the effects of disease or disabilities and to enable regional, country, and subgroup comparisons.

## 2. Materials and Methods 

The cross-sectional survey was conducted between September and November of 2013, and the data were collected using a computer assisted telephone interview system (CATI system). To select the sample, the study employed probability proportional to size (PPS), systematic sampling, and random digit dialing (RDD) of non-institutionalized adults living in private households in Taiwan. It was determined that the representative sample size needed to be at least 1115 with a sampling error of 0.03 based on the 2013 population of two million [15]. After matching the populations of the representative areas, the sample size for this study was 1500. After the respondents completed the survey, the ranking technique was used to weight and adjust for gender and age to ensure representativeness. Before we collected data in the present study, we interviewed 30 adults using face to face and telephone methods to evaluate their AP functioning, and there was an interval of 7–14 days between the two ways to ensure the reliability (inter-rater reliability, ICC). There were not significant differences in the summary score and all domains scores between two ways (*p* > 0.05). Finally, the original sample is 1505, and after weighting for age and gender, the weighted sample number was 1507 (we rounded to take an integer).

### 2.1. The Sampling Design and Participants

To obtain a representative sample of the adult population of Taiwan, two stages were involved. In the first stage, the probability proportional to size (PPS) method was applied to determine the numbers for each subpopulation living in the administrative areas. The systematic random sampling was then applied to obtain a rough sample. In this first stage, the population data were obtained from housing telephone numbers voluntarily registered in the Government Public Telephone Numbers Inventory, a registration system that does not include all of the housing telephone numbers in the area. In the second stage, the last number of the housing telephone numbers was deleted from the rough sample developed in the first stage, and then the random digit dialing (RDD) method was used to identify the real sample. After respondents completed the survey, the ranking technique, which is a weighting method, was used to adjust for gender and age, thus ensuring representativeness of the sample. The sample exclusion criteria included being institutionalized, having a cognitive deficiency, or being unable to communicate [16,17]. This study was approved by the Institutional/Independent Review Board of Medicine, National Taiwan University (IRB No. 201309013) and Buddhist Tzu Chi General Hospital (IRB No. IRB102-92).

Table 1 shows the differences between the weighted sample and the adult population of Taiwan. There were no significant differences in gender, age, or living area compared with the general population of Taiwan (*p* > 0.05). However, 3.3% of those sampled had a disability compared with 4.81% of the adult population in Taiwan having a disability, which constitutes a significant difference (*p* = 0.004).

### 2.2. The Measurement of Activity and Participation Functioning

The instrument used to measure activity and participation was the 36-item Chinese version of the WHO Disability Assessment Schedule 2.0 (WHODAS 2.0) that has been revised as the Functioning Disability Evaluation Scale-adult version (FUNDES-Adult). The FUNDES-Adult was published in 2014 and found to have good validity and reliability [13,18]. These measurement tools were developed based on the WHO’s International Classification of Functioning, Disability and Health (ICF) to measure the level of activity and participation in daily life in the last 30 days with respect to each of the following six domains: (1) cognition (six items)—assessed according to communication and thinking activities such as concentrating, remembering, problem solving, learning, and communicating; (2) mobility (five items)—assessed according to activities such as standing, moving around inside the home, getting out of the home, and walking long distances; (3) self-care (four items)—assessed according to hygiene, dressing, eating, and remaining alone; (4) getting along with people (five items)—assessed by interactions with other people and difficulties that might be encountered within this life domain due to health conditions; (5) life activities (household and school/work, eight items)—assessed according to difficulties with day-to-day activities such as domestic responsibilities, leisure engagements, work, and school tasks; and (6) participation (eight items)—assessed according to social dimensions such as community activities, barriers and hindrances in the environment, and problems with other issues such as maintaining personal dignity. The possible responses for each item include no difficulty, mild difficulty, moderate difficulty, severe difficulty, and extreme difficulty [10,13,18,19]. All domains address both the performance and capability dimensions in this study. The difficulty levels of performance dimensions were judged by the presence or aid of typical assistive technology and personal assistance in real life, while the capability dimensions were evaluated without the aid of devices and personal assistances. Studies on the validity and reliability of FUNDES-Adult have found that the internal consistency of the instrument is excellent (Cronbach’s α ≥ 0.9), and the exploratory factor analysis yields a five-factor FUNDES-Adult structure with a variance of 76.1% and 76.9% and factor loadings of 0.56 to 0.94 and 0.55 to 0.94 for the performance and capability dimensions, respectively. The factor loadings for the second-order confirmatory factor analysis for the performance and capability dimensions range from 0.81 to 0.89 [18].

The difficulty levels were calculated according to the scoring algorithm of WHODAS 2.0, whereas the original scores were converted to 0 through 100 for each domain as well as for the total summary scores, such that higher scores indicated greater levels of difficulty. 

### 2.3. Statistical Analysis

All analyses were performed using IBM SPSS 20.0 (SPSS Inc., Chicago, IL, USA). All scores were converted from the original scores to 0 to 100 for each domain, and to total summary scores based on “item-response-theory” (IRT) scoring [19]. All the psychometric properties of FUNDES-Adult have already been presented in a previous study [13]. 

## 3. Results

### 3.1. Characteristics of the Samples 

Table 1 and Table 2 present the main characteristics of the weighted sample in the present study. Among this sample, 3.3% were people with disabilities and 3.4% were people with a catastrophic illness card who are eligible to receive the copayment exemptions of National Health Insurance in Taiwan. There was a total of 31 types of health conditions with high medical expenses in 2017. Approximately 50% were male, and the average age of the sample was 45.23 years (SD: ±16.83). The majority (68%) of those in the sample were married, and 28.5% were single. The majority of the sample lived in the northern area of Taiwan.

### 3.2. Functioning Scores of the Weighted Sample

Activity and participation scores for the two dimensions and six domains are presented in Table 3. In the performance dimension, the mean of the total summary score was 6.4 (±8.6). The most dramatic performances were exhibited in domain 6, participation, with a mean score of 15.2 (±16.0) and domain 1, cognition, with a mean score of 5.9 (±11.6). With respect to the capability dimension, the mean of the total summary score was 6.7 (±10.0). Again, the two domains exhibiting the most severe functioning were participation, with a mean score of 15.2 (±16.8) and cognition, with a mean score of 5.9 (±11.7). No significant differences were found between gender for any of the domains (*p* > 0.05); domain 2, with a mobility performance score of *p* = 0.03 and a mobility capability score of *p* = 0.02, and domain 6, with a participation capability score of *p* = 0.03. 

There was strong floor effect in most of the domains in both the performance (97.1%) and capability (96.5%) dimensions. This was especially evident in the self-care domain. The floor effect of the total summary score was 21.1% for both dimensions. The ceiling effects were between 0.1 and 1.0% in all domains. The Cronbach’s α of the six domains were between 0.68 and 0.89 in the performance dimension and between 0.74 and 0.91 in the capability dimension.

### 3.3. Activity and Participation Functioning Norms among the General Adult Population

**Figure 1:**
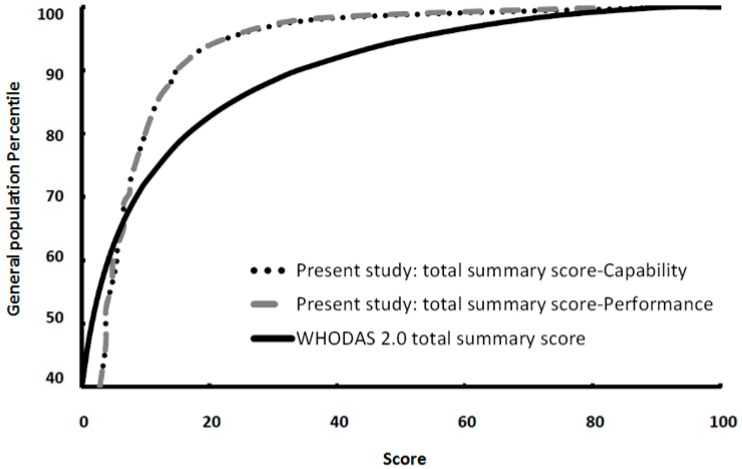
Cumulative frequency curve of the total summary score in the capability and performance dimensions and the WHODAS 2.0 summary total score. Y-axis: cumulative population percentile; X-axis: total summary score. Reference: WHODAS 2.0 total summary score [19].

Figure 1 and Table 4 and Table 5 present the norms for Taiwan’s general adult population. The distribution of the scores was positively skewed, and the scores at the 25th, 50th, and 75th percentiles were 0.94, 3.77, and 8.49, respectively, in the performance and capability dimensions (Table 4). Approximately 90% of the adult population scored less than 15 in both dimensions, while approximately 1% recorded scores above 80 (Table 5).

**Figure 2:**
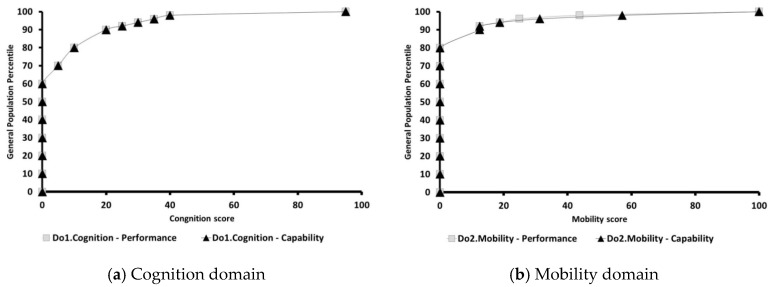

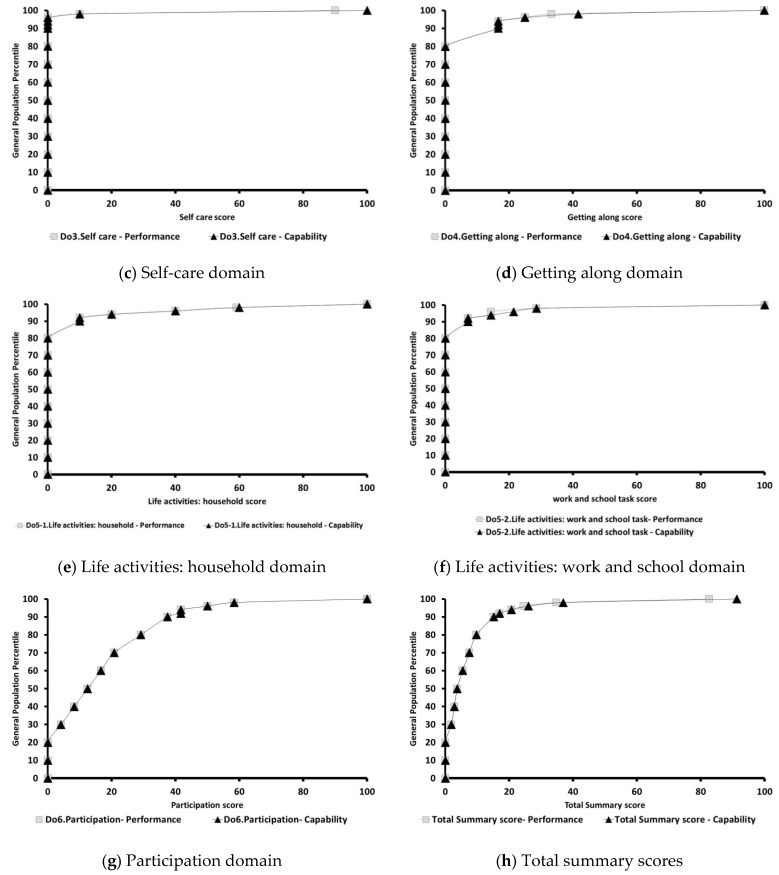
The curves of the functioning scores of the six domains for adults. Population norms for IRT-base scoring of the WHODAS 2.0.

All domain summary scores and percentiles are displayed in Figure 2a–g. Overall, the score distribution curves for performance and capability were similar in every domain. Thus, no significant differences were observed (*p* > 0.05). With respect to the general adult population, the best scores for performance and capability were recorded in domain 3, self-care (Figure 2c), and the worst performance score was found in domain 6, participation (Figure 2g). Performance and capability in the cognitive domain were more serious than in domains 2 through 5 for our adult population, as the cognition functioning score was 0 at the 60th percentile (Figure 2a).

## 4. Discussion

This study aimed to obtain the normative data with respect to the levels of activity and participation using a representative random sample of the general population aged 18 years or older in Taiwan. The FUNDES-Adult survey, which was developed based on the WHODAS 2.0 and the ICF, was administered to this sample by telephone interview. We used the PPS and systematic random sampling methods to obtain a rough sample, and used the weighting method to adjust for gender and age. These methods have been applied in many studies on population norms, such as EQ-5D-3L, SF-36, and other health-related quality of life studies [21,22,23,24]. However, in the present study, our sample of the disability rate was significantly less than the whole population in Taiwan (3.3% vs. 4.81%), and the AP functioning norm was probably overrated (better than real the AP functioning status). Hence, caution is advised with the application of our findings. 

These norms can be used as reference values when comparing the health status or health quality of the different groups in the general population in Taiwan. Because the FUNDES-Adult has excellent correlation with the WHO BRIEF QOL (Taiwan) [13], in July 2012, the disability act in Taiwan mandated that the assessment of individual eligibility for disability benefits be based on the ICF framework, which means that the evaluation must include body function and structure, activities and participation, and environmental and personal factors. The FUNDES-Adult was developed to evaluate an individual’s disabilities [11,13,18]. Table 4 presents the activity and participation norms of a comparison between the general population in the present study and adults with disabilities in Taiwan. Regardless of performance and capability dimensions, the score gap shows a more than 10-fold difference. At the 40th percentile, the functioning score of the performance dimension was 2.8 for the general population, and 37.7 for people with disabilities (Table 5). Thus, there is strong evidence to support reintroducing the activity and participation criteria to the disability eligibility determination system and to allocate the necessary resources to do so. When comparing the results of this study with those of general populations from other countries, the average performance score for other countries, based on the surveys of WHODAS 2.0 full versions, was 0 at the 40th percentile (Table 5) [19]. Thus, the present study indicates that the norm for Taiwan’s population is better than that of other countries. It is possible to infer that the differences in results could be due to the friendly environment and better supports by policy in Taiwan, or to the different data collection and survey methods.

## 5. Conclusions

This is the first cross-national population-based survey to determine the norms for activity and participation for the general population of Taiwan. The norms for the various domains can be used as reference values when comparing the health status of specific groups with the general population, and they can also help explain the performance and capability gaps between groups and populations of other countries. Moreover, the study results will contribute to predicting the needs of people living in Taiwan and in establishing the social support budget for the Taiwan government.

## Figures and Tables

**Table 1:** Comparison of demographic characteristics between the weighted sample and the Taiwan adult population.

Variable	Sample *n* = 1505		Weighted Sample *n* = 1507		Taiwan Adult Population *n* = 19,092,745	Chi-Square Value (*p*-Value)
**Gender**	*n*	%	*n*	%	%	0.014 (0.91)
Male	583	38.7	748	49.7	49.5	
Female	922	61.3	758	50.3	50.5	
**Age (years)**						0.623 (1.00)
18–19	25	1.7	48	3.2	3.4	
20–29	150	10.1	258	17.3	17.2	
30–39	244	16.4	310	20.8	20.6	
40–49	297	20.0	288	19.4	19.3	
50–59	385	25.9	275	18.5	18.5	
60–64	166	11.2	106	7.1	7.1	
65–69	96	6.5	60	4.1	4.1	
70–74	67	4.5	54	3.6	3.6	
75–79	35	2.4	38	2.6	2.7	
80–84	15	1.0	27	1.8	2.0	
≥85	4	0.3	24	1.6	1.5	
**Area**						0.1 (1.00)
Northern	633	42.2	665	44.3	44.7	
Central	460	30.7	369	24.6	24.5	
Southern	363	24.2	414	27.6	27.4	
Eastern	35	2.4	37	2.4	2.4	
Islands	8	0.5	16	1.1	1	
**With Disability**						8.134 (0.004)
Yes	49	3.3	50	3.3	4.81 ^ψ^	
No	1456	96.7	1457	96.7	95.19	

^ψ^ 2013 Taiwan statistics.

**Table 2:** Demographic characteristics of the weighted sample.

Variable	*n*	%
**Marriage (*n* = 1506)**		
Married	1024	68.0
Single	430	28.5
Divorced	15	1.0
Separated	18	1.2
Widowed	19	1.3
**Education (*n* = 1501)**		
Illiterate	32	2.1
Elementary	138	9.2
Junior high	148	9.9
Senior high	408	27.2
Junior college	203	13.5
University	460	30.7
Graduate school	112	7.4
**Disability Type (*n* = 41)**		
Intellectual	2	4.9
Chronic psychosis	10	24.4
Epilepsy	1	2.4
Vision	1	2.4
Hearing	2	4.9
Balance	2	4.9
Loss of functions of primary organs	4	9.8
Physical	19	46.3
**Severity of Disability (*n* = 46)**		
Mild	16	33.6
Moderate	24	52.1
Severe	5	11.2
Profound	1	3.1
**Qualified by New Disability Eligibility System (*n* = 1507)**		
No	1480	98.2
Yes	27	1.8
**Catastrophic Illness Card (*n* = 1505)**		
No	1454	96.6
Yes	51	3.4

**Table 3:** Mean activity and participation scores of the weighted sample.

Dimension and Domain	Domain Score Mean ± SD	Median	Range	Missing (%)
**Performance Dimension (*n* = 1507)**				
Do1. Cognition	5.9 ± 11.6	0.0	0.0~95.0	0
Do2. Mobility	3.4 ± 10.7	0.0	0.0~100.0	0
Do3. Self-care	0.6 ± 4.7	0.0	0.0~90.0	0
Do4. Getting along	3.5 ± 9.8	0.0	0.0~100.0	0
Do5-1. Household	3.6 ± 13.4	0.0	0.0~100.0	0
Do5-2. Work/ school task (*n* = 1017)	1.9 ± 7.7	0.0	0.0~100.0	490 (32.5)
Do6. Participation	15.2 ± 16.0	12.5	0.0~100.0	0
Total Summary Score	6.4 ± 8.6	3.8	0.0~82.6	490 (32.5)
**Capability Dimension (*n* = 1507)**				
Do1. Cognition	5.9 ± 11.7	0.0	0.0~95.0	0
Do2. Mobility	4.0 ± 13.6	0.0	0.0~100.0	0
Do3. Self-care	0.9 ± 6.7	0.0	0.0~100.0	0
Do4. Getting along	3.8 ± 10.7	0.0	0.0~100.0	0
Do5-1. Household	4.1 ± 14.9	0.0	0.0~100.0	0
Do5-2. Work/school task (*n* = 1017)	2.1 ± 8.6	0.0	0.0~100.0	490 (32.5)
Do6. Participation	15.5 ± 16.8	12.5	0.0~100.0	0
Total Summary Score	6.7 ± 10.0	3.8	0.0~91.3	490 (32.5)

**Table 4:** A comparison of the activity and participation norms between the general population and adults with disabilities in Taiwan.

Percentile	Present Study		People with Disabilities [20]	
	Performance	Capability	Performance	Capability
25	0.94	0.94	23.58	26.42
50	3.77	3.77	40.57	45.28
75	8.49	8.49	61.32	69.81

**Table 5:** The percentile score of the total summary score of the WHODAS 2.0 world norm (performance) and two Taiwanese samples, the general population and people with disabilities (capability and performance dimensions).

Percentile	WHODAS 2.0 World Norm	Taiwan General Population		People with Disabilities in Taiwan [20]	
	Performance	Performance	Capability	Performance	Capability
40	0	2.8	2.8	33.7	37.7
46.83	1	3.8	3.8	38.0	42.5
52.08	2	3.8	3.8	41.5	47.2
56.2	3	4.7	4.7	44.6	50.9
59.58	4	4.9	5.4	47.2	53.8
62.46	5	5.7	5.7	50.0	56.6
64.94	6	6.5	6.5	51.9	59.4
67.12	7	6.6	6.6	53.8	61.3
69.05	8	6.6	6.6	55.7	63.2
70.78	9	7.5	7.5	57.5	65.2
72.35	10	7.5	7.5	58.7	67.4
78.42	15	9.4	9.4	65.2	74.5
82.66	20	10.9	10.9	70.8	80.2
85.85	25	12.3	12.3	75.5	84.8
88.35	30	14.1	14.1	79.2	87.7
90.38	35	15.2	15.2	83.0	90.6
94.69	50	21.7	21.7	91.3	96.2
98.14	70	35.9	38	99.1	100.0
99.9	90	81	90.8	100.0	100.0
100	100	82.6	91.3	100.0	100.0
